# Myofunctional assessment for obstructive sleep apnea and the association with patterns of upper airway collapse: a preliminary study

**DOI:** 10.5935/1984-0063.20220030

**Published:** 2022

**Authors:** Fabiane Kayamori, Fabio Augusto Winckler Rabelo, Daniella Nazario, Eric Rodrigues Thuller, Esther Mandelbaum Gonçalves Bianchini

**Affiliations:** 1 Pontifícia Universidade Católica de São Paulo - PUC/SP, Programa de Pós Graduação em Fonoaudiologia - São Paulo - São Paulo - Brazil.; 2 Hospital Samaritano, Departamento de Otorrinolaringologia - São Paulo - São Paulo - Brazil.; 3 EBianchini Fonoaudiologia, Consultório - São Paulo - São Paulo - Brazil.; 4 Hospital Sírio-Libanês, Instituto Sírio-Libanês de Ensino e Pesquisa - São Paulo - São Paulo - Brazil.

**Keywords:** Sleep Apnea, Obstructive, Examination, Physical, Therapy, Speech

## Abstract

**Objectives:**

To organize an assessment instrument with questionnaires and myofunctional orofacial/oropharyngeal assessment for OSA patients and correlate it with the upper airway obstructive site detected during drug-induced sleep endoscopy (DISE).

**Material and Methods:**

29 OSA patients aged 22-65 years with an indication to undergo DISE to evaluate an alternative treatment to PAP and signed the consent form. Patients over 65 years old with maxillofacial deficiency and BMI>30 were excluded. The subjects answered the Pittsburgh, Berlin (snore), and Epworth questionnaires. The myofunctional orofacial/oropharyngeal assessment comprised soft palate, palatine pillars, and uvula (structure and mobility), tonsils (size), mandible (bony bases), hard palate (depth and width), tongue (posture, volume, width, and height), floor of mouth (mylohyoid), tongue suction and sustaining (mobility), “lowering of the back of the tongue” (stimulus), which were scored by three speech-language pathologists with expertise. DISE was scored according to VOTE classification. The statistical analysis (t-test) compared groups without and with obstruction in VOTE with questionnaires and myofunctional orofacial/oropharyngeal assessment.

**Results:**

The following were significantly different: snoring frequency (p=0.03) with VOTE/velopharynx; intensity (p=0.02) and frequency of snoring (p=0.03) with VOTE/lateral wall of oropharynx; suction the tongue and sustain (p=0.02) with VOTE/velopharynx; hard palate depth (p=0.02) and width (p=0.05) with obstruction VOTE/epiglottis; tonsils volume (p=0.05) with VOTE/epiglottis; tongue posture (p=0.00) with obstruction VOTE/epiglottis; floor of the mouth (p=0.02) with VOTE/epiglottis.

**Conclusion:**

Higher snoring frequency and intensity was observed in patients with obstruction at the velopharynx and oropharyngeal lateral wall. Obstruction at the velopharynx was associated with poor tongue ability to suck the tongue against the hard palate. Obstruction at the epiglottis had structural and functional associations, including the oropharyngeal lateral wall, affected by the palatine tonsils size, depth and width of the hard palate, tongue position, and flaccidity of the floor of mouth. Considering that this is a preliminary study, the data should be carefully verified and not generalized.

## INTRODUCTION

Obstructive sleep apnea (OSA) is a condition in which breathing is partially or fully interrupted during sleep. The prevalence of individuals with moderate and severe disease in the general population ranges from 6 to 17% in a systematic review^1^, which increases the risk of metabolic and cardiovascular diseases^2^ and compromises the quality of life^3^. An epidemiological study performed in São Paulo, with 1,041 volunteers showed a prevalence of mild OSA at 21,7% and moderate/severe OSA at 24,8% in male individuals^4^. The prevalence of mild OSA in female subjects was at 20,9%, and moderate/severe OSA was at 9,6%^4^. Polysomnography is the standard diagnostic test that offers several relevant information about sleep quality and quantifies breathing events^5^. However, it does not assess the site of upper airway (UA) collapse.

Positive airway pressure (PAP) is usually the first treatment option, providing UA collapse resolution and significantly decreasing the disease burden. It improves sleepiness, blood pressure, and sleep-related quality of life^6^. However, adherence to the treatment has been an issue despite the efforts towards behavioral intervention and coaching^7^, which encourages patients to try to find other treatment options^8^.

The speech-language pathologist (SLP) approach using orofacial and oropharyngeal myofunctional therapy emerged as another option to treat OSA^9^. Although some assessment protocols have been developed to direct that specific therapy, only one of them has been published^10^. The *expanded protocol of orofacial myofunctional evaluation with scores* was tested for validity, reliability, and psychometric properties in subjects with and without OSA^10^. Despite being effective, the protocol is not highly specific regarding the anatomical and functional variables of OSA to help assess patient’s risks of having OSA or even to target myofunctional therapy specific to that problem.

The pathophysiology of OSA is multifactorial and complex, and evidence suggests its treatment must be personalized according to the different phenotypes^11^. There is no doubt that craniofacial features, such as mandibular retropositioning, maxillary deficiency, and lower displacement of the hyoid bone, are associated with a reduced pharyngeal area, which increases the prevalence of OSA. In the same way, soft tissue hypertrophy, such as larger tonsils, tongue, lateral pharyngeal walls, and soft palate, also reduces the pharyngeal area and contributes to disease severity^11,12^. However, the upper airway with no significant anatomical findings is common in OSA patients, highlighting the other pathophysiological mechanisms and the importance of differentiating OSA phenotypes.

Drug-induced sleep endoscopy (DISE) is a test that provides a dynamic assessment with direct visualization of the UA during apnea episodes, enabling the detection of the obstruction site in a muscular relaxation condition similar to spontaneous sleep^13^. This test may allow treatment customizing based on the respective obstruction site and pattern, positively impacting the outcome^14,15^. However, DISE requires standardization to obtain reliable information, which depends on the used sedation protocol^16^, encouraging the search for easier methods to predict the site and type of UA collapse.

Orofacial and oropharyngeal clinical assessment instruments provide detailed descriptive data on the structures and their functional behavior. However, there is no specific instrument for orofacial and oropharyngeal analysis that can predict the presence of sleep-disordered breathing nor the site of collapse involved. Thus, there is room to describe a myofunctional orofacial and oropharyngeal phenotype based on clinical assessment data that can help predict the upper airway obstructive site during DISE. Thus, using a non-invasive low-cost assessment instrument may allow a more assertive treatment selection for OSA patients.

## OBJECTIVES

This study aimed to organize an assessment instrument with questionnaires and myofunctional orofacial and oropharyngeal assessment for OSA patients and correlate it with the upper airway obstructive site detected during drug-induced sleep endoscopy (DISE).

## MATERIAL AND METHODS

The human research ethics committee approved this project under the certificate of presentation for ethical consideration No. CAEE: 63408516.8.0000.5482.

This study only included male patients trying to find a PAP alternative treatment due to low adherence to such therapy. The subjects were referred to undergo DISE to detect the upper airway obstructive site. Patients over 65 years of age who were obese (BMI>30) and had maxillofacial deficiency or deformities and any type of neurological disease or clinical conditions that might interfere in the research protocol’s fulfillment were excluded.

After ethical research procedures, 29 adults (23-65 years old) diagnosed with OSA were included. The subjects were submitted to an ENT clinical evaluation, and the physician recommended DISE to detect the obstructive site before proposing the best treatment option for the patient. All patients voluntarily consented to participate in this study in conformity with the ethical protocol.

Due to the risks involving sedation, DISE was not proposed to any subject without OSA. Those with no obstructions during DISE were considered the control group.

### Procedures

Immediately before DISE, all subjects answered questionnaires (Pittsburgh sleep quality index, Berlin questionnaire for snoring, Epworth sleepiness scale) and underwent a myofunctional assessment for OSA as described below.

### Pittsburgh sleep quality index

The Pittsburgh sleep quality index was applied to assess the sleep quality according to the following parameters: subjective sleep quality, sleep latency, duration of sleep, normal sleep efficiency, sleep disorders, sleep medication use, and daytime fatigue^17^. The higher the score, the worse the sleep quality.

### Snoring questionnaire - Berlin questionnaire

Snoring was evaluated regarding its frequency and intensity according to the proper segment of the Berlin questionnaire^18^ for the snoring intensity. A scale from 1 to 3 was used, where (1) is as high as breathing, (2) is as high as speaking, and (3) is so loud one can hear from another room.

For the frequency, a scale of 0 to 4 was used, where (0) is never or rarely, (1) is one or two times a month, (2) is one or two times a week, (3) is three or four times a week, and (4) is almost every day.

### Epworth sleepiness scale (ESS)

Daytime sleepiness was assessed with the Epworth sleepiness scale (ESS), a simple, self- administered questionnaire that measures patients’ daytime sleepiness levels. The chance of dozing off is ranked from 0 to 3, being (0) none, (1) a small chance of dozing off, (2) a moderate chance of dozing off, and (3) a high chance of dozing off. It assesses specific situations such as while sitting and reading; watching television; sitting in public spaces (movies, church, waiting room); as a passenger of train, car, or bus during a one-hour nonstop ride, lying down to get some rest during the afternoon; talking to someone; sitting peacefully after lunch (with no alcohol); driving the car while waiting for a few minutes in traffic. ESS score up to 10 points is considered normal, and above 10 is considered excessive daytime sleepiness^19^.

### Myofunctional assessment for OSA

The development of the proposed myofunctional assessment was based on a previously described myofunctional orofacial assessment in an existing PhD thesis^20^ containing Friedman classification^22^ (which involves the positioning of the tongue, tonsil size, besides the palatine pillars)^23^ was added floor of mouth evaluation^24^ and made adaptations in order to develop the current protocol. The myofunctional assessment was performed, photographed, and recorded by the same speech-language pathologist (SLP) examiner, who has experience in OSA cases and is accredited in sleep medicine by the Brazilian Association of Sleep Medicine (ABS). The UA structures were evaluated under rest conditions, during mobility, and under stimulus. The complete test also included facial photos: frontal, right side, and left side; videos of all functional or response to stimulus situations; anthropometric data: weight, height, cervical circumference, and abdominal circumference.

**Figure 1:**
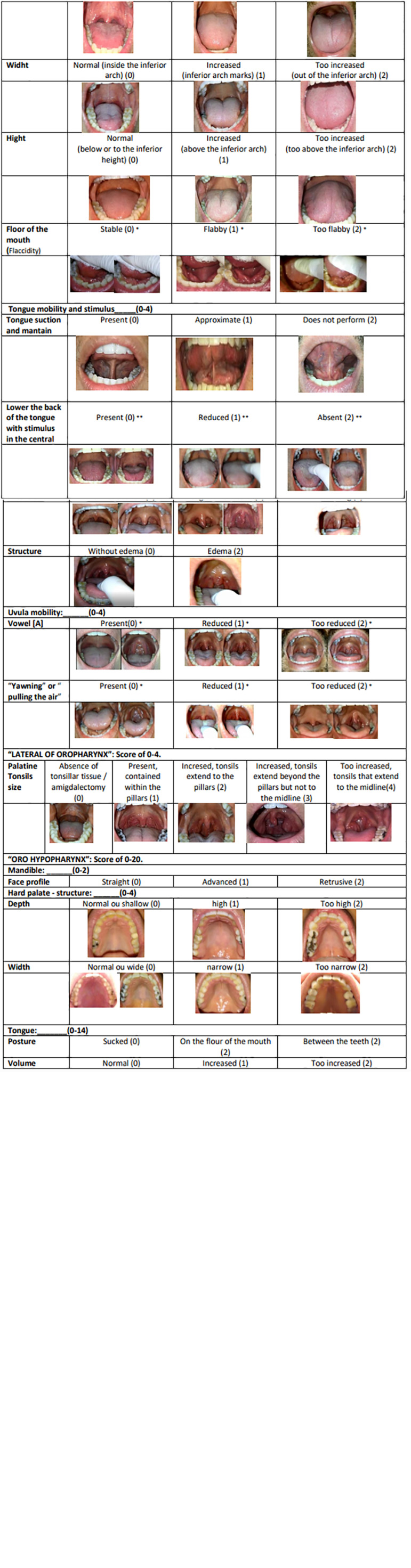
The structures initially described were related to the “**velopharynx**”, the minimum and maximum score being respectively 0-20.

The intraoral examination included myofunctional assessment related functional and structural aspects and was based on the same concept used in VOTE (velum, oropharynx lateral wall, tongue base, and epiglottis) classification for the sleep endoscopy findings: “**velopharynx**,” **“lateral of the oropharynx,”** and **“oro-hypopharynx.”** Each assessed structure was scored for classification, where the lower the score, the better the structure, as detailed in Figure 1.

The structures related to the **“velopharynx”**, were described, the minimum and maximum score respectively 0-20.

SOFT PALATE STRUCTURE: the soft palate was analyzed for its extension and rated 0-2.

SOFT PALATE MOBILITY: the soft palate was analyzed for its mobility during the [A] vowel production and “yawning” or “pulling the air”, rated 0-4.

STRUCTURE OF THE PALATINE PILLARS: palatine pillars were analyzed for their transverse distance and the presence of web palate, rated 0-2.

PALATINE PILLARS MOBILITY: transverse opening mobility of the palatine pillars was analyzed during the [A] vowel production and “yawning” or “pulling the air”, rated 0-4.

UVULA STRUCTURE: the uvula was analyzed for its extension/size and according to the presence or absence of edema, rated 0-4.

UVULA MOBILITY: the uvula was analyzed for its mobility according to the elevation, reflecting the muscle contraction during the [A] vowel production and during “yawning” or “pulling the air”, rated 0-4.

The structures related to the **“lateral of oropharynx”** were described, the minimum and maximum score respectively 0-4.

PALATINE TONSILS: analyzed according to their size and rated 0-4.

The structures related to the “**oro-hypopharynx**”, the minimum and maximum score respectively 0-20.

MANDIBLE: the mandible was analyzed from its lateral side considering its position regarding the other bone base/to the maxillary bone, rated 0-2.

HARD PALATE: the hard palate was analyzed for depth and width, rated 0-4.

The bone structures (hard palate and mandible) were analyzed and scored. Such structures define the tongue area and were included in the oro-hypopharynx item. The deficiency of these structures, transverse and anteroposterior, compromise tongue accommodation and posture, affecting its stability during sleep.

TONGUE: tongue structural and mobility analysis were rated 0-14, adding the results of the items described below:

Normal tongue posture: after the patient was guided to notice where his tongue was located, he was asked where his tongue’s anterior part was placed.

Volume (transversal and horizontal) within the oral cavity: association between tongue width and height regarding the inferior arch.

Floor of mouth (mylohyoid muscle) was rated after the patient was asked to push the tip of his tongue up against the examiner’s index finger, placed in the anterior part of the tongue to create resistance, and always enabling a clear view of the floor of the mouth in its anterior region.

Mobility in two situations: during tongue suction against the hard palate and sustaining; lowering the back of the tongue with a stimulus in the central area of the tongue and sustaining to enable visualization of the oropharynx.

This assessment data was analyzed by three judges who were SLPs with more than 10 years of experience in assessing patients with myofunctional orofacial disorders, sleep apnea, and snoring. They were blinded to the questionnaires and DISE results.

Each subject’s data were analyzed separately by each of the three SLPs, starting from a previously defined score. In case there were any divergences after gathering the analysis result, a second analysis stage was performed to reach an agreement.

### Drug-induced sleep endoscopy (DISE)

Drug-induced sleep endoscopy (DISE) was performed using only propofol infused with a target-controlled pump by an experienced anesthesiologist following the protocol described by Rabelo et al. (2013)^13^, which was able to reproduce the AHI from a control PSG. The sedation level was monitored using the bispectral index system (BIS), which was kept between 60 and 70 during the period recorded for posterior analysis.

The DISE findings were described using the VOTE classification system, which divides the upper airway into four areas to evaluate the collapse, velopharynx, oropharynx, tongue base, and epiglottis^25^.

Velopharynx: the collapse occurs due to the structures of the soft palate, uvula, and lateral pharyngeal wall. This type of collapse can happen with anteroposterior, lateral, or concentric configuration.

Oropharynx: the collapse occurs mainly due to the palate tonsils and oropharyngeal lateral walls (muscles and parapharyngeal fat). This type of collapse only happens on a lateral configuration.

Tongue base: the collapse occurs by relaxation and posterior displacement of the tongue or tissue hypertrophy, mainly the lingual tonsillar tissue. This type of collapse is anteroposterior.

Epiglottis: the primary collapse of the epiglottis can be anteroposterior, caused by the inflection of the central epiglottis area; secondary to the tongue base^26^ or lateral, caused by cartilage weakness that enables such folding^25^.

The obstruction degree is rated based on the following criteria^25^: zero (0) - no vibration or obstruction; one (1) - partial obstruction or vibration; two (2) - complete obstruction; (x) - not visualized.

### Statistical analysis

The data were analyzed using the statistical package SPSS for Windows, version 20, IBM Corporation, and Minitab 16.

The normal distribution for the continuous variable was examined using the Kolmogorov-Smirnov test. According to the statistical analysis, the study subjects were only men, and according to Tables 2 and 4 they have a normal distribution, except for age.

**Table 2:** Descriptive data on the frequency of the types of obstruction, degree of obstruction and configuration of obstruction according to DISE exam and VOTE classification.

STRUCTURE	(N=29)	Degree of obstruction	CONFIGURATION		
			**ANTERO POSTERIOR**	**LATERAL**	**CONCENTRIC**
VELUM	27	24 total/3 partial	19 (70.4%)	1 (3.7%)	7 (25.9%)
OROPHARINX LATERAL WALLS	14	12 total/2 partial	___ _	14 (100%)	___ _ _
TONGUE	21	20 total/1 partial	21 (100%)	___ __	___ _ _
EPIGLOTTIS	7	5 total/2 partial	7 (100%)	___ __	___ _ __

**Table 4:** Comparison (t-test) among age data, anthropometric data and questionnaires in patients with obstruction and without obstruction in VOTE.

	VOTE Velopharynx (N=29)			VOTE OLW (N=29)			VOTE Tongue (N=29)			VOTE Epiglottis (N=29)		
	**WTO (5)**	**WO (24)**	**p**	**WTO (17)**	**WO (12)**	**p**	**WTO (9)**	**WO (20)**	**p**	**WTO (5)**	**WO (24)**	**p**
Age (years)	36.4±6.5	43±10.4	0.18	41.8±10.6	42.0±9.7	0.94	40.3±6.0	42.7±11. 5 (20)	0.56	44.8±9.3	41.3±10,3	0.49
BMI (kg/m^2^)	27.4±2.1	27.9±3.2	0.73	27.6±3.0	28.1±3.1	0.67	27.0±3.3	28.2±2.8	0.34	27.3±3.3	28.0±3,0	0.64
CC (cm)	40.4±1.8	41.4±2.5	0.41	41.1±2.5	41.5±2.4	0.63	41.2±2.7	41.3±2.4	0.99	40.9±2.0	41.3±2.5	0.76
AC (cm)	101.2±7	98.3±9.8	0.54	98.6±9.9	99.1±9.0	0.88	97.2±9.3	99.5±9.5	0.55	101.7±10 .7	98.2±9.2	0.46
Pittsburgh	5.2±2.8	6.5±2.8	0.33	6.1±2.8	6.6±2.8	0.66	6.1±2.0	6.4±3.1	0.80	5.8±2.2	6.4±2.9	0.66
Berlin (int)	2.8±0.8	3.3±1	0.37	2.8±1.1	3.7±0.5	0.02*	3.3±1.0	3.1±1.0	0.57	2.6±1.1	3.3±1.0	0.16
Berlin (freq)	3.2±0.8	3.8±0.5	0.03*	3.5±0.7	4.0±0.0	0.03*	4.0±0.0	3.6±0.7	0.09	3.6±0.9	3.7±0.5	0.62
Epworth	11.6±6.3	9.5±4.2	0.34	10.2±4.7	9.3±4.4	0.57	8.6±4.1	10.4±4.7	0.32	8.0±5.3	10.2±4.3	0.33

Notes: OLW = Oropharynx lateral walls; WO = With obstruction; WTO = Without obstruction; BMI = Body mass index; Kg/m2 = Kilogram per square meter; CC = Cervical circumference; AC = Abdominal circumference; cm = Centimeters; int = Intensity; freq = Frequence; p=significance -

* p<0,05.

The comparative analysis (t-test) was used to compare groups with and without obstruction in the VOTE classification according to age, anthropometric, questionnaires, and myofunctional assessment data. A *p*-value of 0.05 was considered.

Due to the sample size, no other statistical analyses were possible.

## RESULTS

The total patients who fulfilled the criteria were 29 male subjects (23-65 years old) with an average of 41 years. According to the statistical analysis (Table 1), the sample has a normal distribution, except for age. The data regarding the variables of the subject’s analyses are shown in Table 1.

**Table 1:** Descriptive data of the subjects.

Descriptive data	
N=29	Mean, SD
Age (years)	41.9±10.0
BMI (Kg/m2)	27.8±3.0
CC (cm)	41.2±2.4
AC (cm)	98.8±9,3
Pittsburgh (sc)	6.3±2.75
Berlin (int) (sc)	3.2±1.0
Berlin (freq) (sc)	3.7±0.6
Epworth (sc)	9.8±4.52

SD=standard deviation; BMI=body mass index; Kg/m2=kilogram per square meter; CC=cervical circumference; AC=abdominal circumference; cm=centimeters; sc=score.

The descriptive data on the frequency of the obstruction types, degree, and configuration according to the DISE exam and VOTE classification is shown in Table 2.

Descriptive data of the obstruction type and degree detected in the 29 subjects, considering that each subject may have more than one obstructive site, showed that the velum was the most common site of obstruction (93%), followed by the tongue (72%), oropharynx lateral walls (48%), and epiglottis (2,4%) (Table 2).

The data regarding the myofunctional assessment for OSA scores of the subjects analyzed in the study are shown in Table 3.

**Table 3:** Data of the myofunctional assessment for OSA scores.

Myofunctional Assessment	
**N=29**	**Mean ± SD**
**MA Velopharynx (sc)**	8.1±4.28
Soft palate structure: extension	1±0.65
Soft palate mobility: say A	0.92±0.78
Soft palate mobility: yawning	0.3±0.61
Palatine pillars structure pillars	1.12±0.74
Palatine pillars mobility: say A	1.15±0.8
Palatine pillars mobility yawning	0.73±0.8
Uvula structure: extension	0.57±0.73
Uvula structure: structure	0.3±0.7
Uvula mobility: say A	1.23±0.82
Uvula mobility: yawning	0.73±0.8
MA lateral of oropharynx (sc)	1.26±0.95
Palatine Tonsils size	1.26±0.95
**MA oro hypopharynx (sc)**	9.26±4.09
Mandible: face profile	0.88±0.97
Hard palate structure: depth	0.65±0.75
Hard palate structure: width	0.58±0.73
Tongue structure: posture	1.15±0.98
Tongue structure: volume	1.07±0.80
Tongue structure: width	1.08±0.7
Tongue structure: hight	1.23±0.80
Tongue structure: floor of mouth	1.15±0.61
Tongue mobility: sucking the tongue and sustaining	0.42±0.50
Tongue mobility: lower the back after stimulus	1.04±0.94
**TMA (sc)**	18.61±7.04

SD=standard deviation; MA = Myofunctional Assessment; TMA = Total Myofunctional Assessment.

Table 4 shows the comparison among age and anthropometric data and questionnaires in patients with and without obstruction in VOTE.

The data comparing the variable analyzed with the obstructive site accordingly to the VOTE classification had a statistically significant difference in the frequency section of the Berlin questionnaire (*p*=0.03) when comparing the groups with and without obstruction at the velopharynx, which suggests that the groups with obstruction at this site had higher snoring frequency. The group with obstruction at the lateral wall of the oropharynx also had a statistically significant difference in the Berlin questionnaire, both in frequency (*p*=0.02) and intensity (*p*=0.03), when compared with the group without obstruction, indicating that the group with obstruction at this site has higher snoring frequency and intensity (Table 4).

Table 5 compares the myofunctional assessment scores for patients with obstruction and without obstruction in VOTE.

**Table 5:** Comparison (t-test) of the myofunctional assessment scores for patients with obstruction and without obstruction in VOTE.

	VOTE velopharynx (N=29)			VOTE OLW (N=29)			VOTE Tongue (N=29)			VOTE Epiglottis (N=29)		
	**WTO** **(5)**	**WO** **(24)**	**p**	**WTO** **(17)**	**WO** **(12)**	**p**	**WTO** **(9)**	**WO** **(20)**	**p**	**WTO** **(24)**	**WO** **(5)**	**p**
**MA** **Velopharynx**	7.2±6.5	8.2±3.8	0.65	7.1±4.7	9.3±3.7	0.19	7.9±3.1	8.1±4.8	0.93	8.1±4.2	7.6±5.0	0.82
**MA Lateral Oropharynx**	1.4±1.5	1.2±0.8	0.69	1.1±0.8	1.5±1.1	0.23	1.2±0.7	1.3±1.1	0.94	1.1±0.8	2.0±1.4	0.05*
**MA Oro- Hypopharyn x**	9.0±5.3	9.5±3.9	0.79	9.7±4.6	9.1±3.5	0.69	8.9±3.5	9.7±4.4	0.63	10.0±4.1	6.8±3.3	0.11
**TMA**	17.6±10.9	18.9±6.3	0.71	17.8±8.3	19.8±4.8	0.47	18.0±5.2	19.0±7.8	0.73	16.4±8.9	19.2±6.7	0.43

Notes: OLW = Oropharynx lateral walls; WO = With obstruction; WTO = Without obstruction; MA = Myofunctional assessment; TMA = Total myofunctional assessment; p = Significance -

* p<0,05.

According to the VOTE classification, the comparison between the myofunctional assessment for OSA scores with the obstructive site showed a statistically significant difference in the myofunctional assessment of the oropharynx lateral walls (*p*=0.05) when comparing the groups with and without obstruction at the epiglottis. That indicates that the groups with obstruction at this site presented an enlarged palatine tonsil (higher score) (Table 5).

Table 6 shows a comparison between the myofunctional assessment structures for OSA with the obstructive site according to the VOTE classification.

**Table 6:** Comparison among (t-test) structures of the myofunctional assessment in patients with obstruction or without obstruction in VOTE.

N=29	VOTE Velopharynx (N=29)			VOTE OLW (N=29)			VOTE Tongue (N=29)			VOTE Epiglottis (N=29)		
	WTO (5)	WO (24)	p	WTO (17)	WO (12)	P	WTO (9)	WO (20)	p	WTO (24)	WO (5)	p
**Soft palate structure: extension**	0.8±0.5	1.0±0.7	0.46	0.9±0.7	1.2±0.6	0.26	0.8±0.7	1.1±0.6	0.23	1.1±0.6	0.6±0.6	0.14
**Soft palate mobility: say A**	0.6±0.9	1.04±0.7	0.26	0.8±0.8	1.3±0.8	0.10	1.2±0.8	0.9±0.8	0.24	1.0±0.8	0.8±0.8	0.61
**Soft palate mobility: yawning**	0.4±0.6	0.3±0.6	0.83	0.3±0.6	0.4±0.7	0.61	0.4±0.7	0.3±0.6	0.57	0.3±0.6	0.4±0.6	0.83
**Palatine pillars structure pillars**	0.6±0.9	1.3±0.7	0.07	1.1±0.9	1.3±0.7	0.51	1.4±0.5	1.0±0.8	0.14	1.0±0.8	1.6±0.6	0.13
**Palatine pillars mobility: say A**	0.8±1.1	1.3±0.7	0.26	1.0±0.8	1.5±0.7	0.06	1.6±0.7	1.0±0.8	0.09	1.2±0.8	1.0±1.0	0.61
**Palatine pillars mobility yawning**	0.6±0.9	0.8±0.8	0.71	0.7±0.8	0.8±0.8	0.55	0.6±0.7	0.8±0.8	0.46	0.8±0.8	0.6±0.9	0.71
**Uvula structure: extension**	0.8±0.9	0.5±0.7	0.48	0.5±0.6	0.8±0.9	0.32	0.3±0.7	0.7±0.7	0.22	0.6±0.7	0.6±0.9	0.96
**Uvula structure: structure**	0.4±0.9	0.3±0.7	0.33	0.3±0.7	0.3±0.8	0.66	0.0±0,0	0.4±0.8	0.80	0.25±0.7	0,4±0.9	0.66
**Uvula mobility: say A**	1.0±1.0	1.2±0.8	0.76	1.0±0.8	1.3±0.8	0.43	1.1±0.9	1.1±0.8	0.97	1.1±0.8	1.0±1.0	0.76
**Uvula mobility: yawning**	1.2±0.8	0.6±0.8	0.12	0.8±0.8	0.5±0.8	0.30	0.4±0.5	0.8±0.9	0.28	0.7±0.8	0.6±0.9	0.79
**Tonsils**	1.4±1.5	1.2±0.8	0.69	116±0.8	1.5±1.1	0.23	1.2±0.7	1.3±1.1	0.94	1.1±0.8	2.0±1.4	0.05*
**Mandible: face profile**	1.0±1.0	0.8±1.0	0.67	1.1±1.0	0.4±0.8	0.05	0.3±0.7	1.1±1.0	0.06	0.9±1.0	0.4±0.9	0.29
**Hard palate structure: depth**	0.6±0.9	0.8±0.7	0.69	0.8±0.8	0.6±0.6 7	0.41	0.4±0.5	0.9±0.8	0.18	0.9±0.7	0.0±0.0	0.02*
**Hard palate structure** **width:**	0.4±0.9	0.6±0.7	0.54	0.6±0.8	0.6±0.7	0.99	0.7±0.9	0.6±0.7	0.70	0.7±0.8	0.0±0.0	0.05*
**Tongue structure: posture**	0.8±1.1	1.3±1.0	0.28	1.1±1.0	1.5±0.9	0.24	1.1±1.1	1.3±1.0	0.64	1.5±0.9	0.0±0.0	0.00*
**Tongue structure: volume**	1.4±0.9	1.0±0.8	0.32	1.1±0.9	1.1±0.7	0.94	1.1±0.8	1.05±0.83	0.85	1.0±0.8	1.6±0.6	0.10
**Tongue structure: width**	1.4±0.9	1.0±0.7	0.26	1.1±0.8	1.0±0.6	0.67	1.0±0.7	1.10±0.72	0.73	1.0±0.7	1.4±0.6	0.26
**Tongue structure: hight**	1.4±0.9	1.3±0.8	0.71	1.2±0.9	1.4±0.7	0.44	1.3±0.7	1.3±0.9	0.80	1.2±0.8	1.6±0.6	0.33
**Tongue structure: floor of mouth**	0.8±1.1	1.3±0.5	0.11	1.1±0.7	1.3±0,5	0.37	1.3±0.5	1.2±0.7	0.47	1.3±0.6	0.6±0.6	0.02*
**Tongue mobility: sucking the tongue and sustaining**	0.0±0.0	0.6±0.5	0.02*	0.5±0.5	0.5±0,5	0.88	0.7±0.5	0.4±0.5	0.20	0.5±0.5	0.2±0.5	0.18
**Tongue mobility: lower the back after stimilus**	1.2±1.1	0.9±0.9	0.55	1.2±1.0	0.7±0.9	0.16	0.9±0.9	1.0±1.0	0.78	1.0±1.0	1.0±1.0	0.93

Notes: V = VOTE; OLW = Oropharynx lateral walls; WO = With obstruction; WTO = Without obstruction; p = Significance -

* p<0,05.

The result showed a statistically significant difference in assessing several variables when comparing the groups with and without obstruction at the epiglottis. Enlarged tonsils (higher score for tonsils) were associated with more obstruction at that site (*p*=0.05). Additionally, there was a statistically significant difference in depth (*p*=0.02) and width (*p*=0.05) of the hard palate, indicating that higher and narrower hard palate (higher score) can also be associated with obstruction at the epiglottis. Tongue position was also examined, and there was a significant statistical difference in the assessment of the normal tongue position (*p*=0.00), suggesting that the tongue positioned on the floor of mouth (higher score) was associated with more obstruction of the epiglottis. Even the floor of mouth showed a significant difference (*p*=0.02), and when unstable (higher score) was associated with more obstruction at the epiglottis.

Regarding the other obstructive sites detected during DISE, only the comparison between the groups with and without obstruction at the velopharynx showed a statistically significant difference (*p*=0.02) of the tongue mobility in sucking and sustaining against the hard palate. In this sample, the group with obstruction at that site presented less tongue mobility and more difficulty performing the tasks (higher score). Otherwise, better tongue mobility in sucking and sustaining (lower score) was associated with less obstruction at the velopharynx (Table 6).

Considering that this is a preliminary study, the data should be carefully verified and not generalized.

## DISCUSSION

To this date, this preliminary study seems to be the first that tried to associate data from a myofunctional assessment with the sites of collapse observed during DISE according to the VOTE classification.

Based on previous assessments performed in randomized studies related to primary snore and OSA^20,21^, this assessment proposed a more specific and detailed approach that included mobility of the orofacial and oropharyngeal structures, seeking to guide the myofunctional therapy for OSA. Compared to the VOTE classification, this assessment allowed us to think about the physiology and function of the soft tissues and other anatomical structures during sleep, which helped clarify the possible effects of the anatomic structures that a specific myofunctional assessment can evaluate.

This protocol can be used to evaluate OSA patients, directing the therapy according to soft tissue characteristics, as previously described in patients with OSA^27^, also helping to predict limitations and offering guidance to referrals to other treatments^28^. It could also help evaluate orofacial myofunctional therapy according to modifications measured by MRI after therapy on moderate OSA patients post-stroke, such as the soft palate length reduction^29^.

Except by age, the descriptive data had a small standard deviation, but there were no differences in the general or specific myofunctional assessment or DISE findings, even though previous studies have found differences in elderly people^30,31^. This study selection criteria, which excluded patients older than 65, or its sample size may have contributed to such a result.

This study showed that the obstruction percentage in each site is fairly similar to the findings in the literature, in which the most frequent site of obstruction was the velopharynx followed by the retroglossal area^32^. Although obesity is a risk factor for OSA and research has proved that subjects with BMI over 30 have a greater probability of multilevel obstruction^33^, this study found no correlation of such variable with the degree of upper airway obstruction detected during DISE, highlighting the fact that the BMI in our sample was under 28.

Snoring is the variable with the most statistically relevant data in this study and is more frequent in the group with velopharyngeal and oropharyngeal lateral wall obstruction and even more intense in the second group. Such outcome suggests that the velopharynx and oropharyngeal lateral walls were involved in the vibratory mechanism causing snoring and can potentially guide the treatment strategies in patients with primary snoring^34,35^. A different study that recorded snoring and evaluated patients with DISE found that the sound frequency was correlated with the anatomic structures involved, which suggests the snoring characteristics may be useful to predict the obstructive sites and surgical responses^36^. Despite that, snoring intensity and frequency were not correlated with the myofunctional assessment of the palatine velum and oropharyngeal lateral walls, reinforcing the hypothesis that muscle relaxation and other mechanisms may be involved in snoring.

Regarding the association between the myofunctional assessment score and the DISE findings, there was a statistically significant difference between the ability to suck the tongue and sustain it against the hard palate and the obstruction detected at the velopharynx. In that case, individuals with an increased ability had less obstruction at that site. Tongue posture is supposed to be maintained by its attachment to the hard palate, sustained by the negative pressure generated from the suction, consequently stabilizing the soft palate. It is believed that this mechanism also enables the stability of the velopharyngeal area^37^, and so the tongue-repositioning maneuver was able to stabilize the tongue when a nasal breathing pattern was present. Myofunctional therapy reestablishes correct tongue posture and its physiological function, which can be trained with specific exercises such as sucking the tongue upward against the hard palate, and has shown improved snoring and OSA after treatment^9,38,39^. However, being able to suck the tongue depends on the motor ability and the hard palate transverse and vertical dimensions.

The obstruction at the epiglottis during DISE is the variable that showed the most statistically significant association with the structures evaluated in the myofunctional assessment, starting with the oropharyngeal lateral wall results, affected by the palatine tonsils size, which were associated with the occurrence of epiglottic obstruction. That anatomical structure, whose size contributes to the obstruction of the upper airway^40^, can create enough negative pressure leading to the collapse of the epiglottis. Following that rationale, surgical removal of the hypertrophied tonsils may be advised even in cases with epiglottic collapse once such surgical option has already been established in the OSA surgical treatment^41^. Additionally, there was a statistically significant difference in the hard palate depth and width, implying a higher and narrower hard palate, suggesting that the change in tongue position, initially on the mouth floor, can also be associated with obstruction at the epiglottis. On the other hand, the more stable floor of mouth was associated with reduced obstruction at the epiglottis. Although these data seem remarkably interesting, they cannot be generalized due to the small sample size, especially concerning the epiglottis collapse.

We hypothesize that a stable floor of mouth offers tongue support, maintaining the hyoid bone position and preventing the epiglottic collapse^26^. Similarly, the higher and narrow hard palate would compromise the adequate tongue attachment with a tendency to change the hyoid position and cause epiglottic obstruction. Thus, subjects with better tongue suction have a better rest tongue position and are less prone to epiglottic collapse, as previously proposed^26^. However, the correlation between the depth and width of the hard palate position of the tongue and sustained floor of mouth with the velopharyngeal, oropharyngeal, and tongue base obstruction are contradictory and opposes to this theory, highlighting that the collapse is not only dependent on the anatomy but also of neuromuscular mechanisms^42,43^.

Hypothetically, the wider palatine pillars or palatal web^23,44^ indicate narrower structures that contribute to a higher chance of collapse at the velopharynx, but our data has only shown an association tendency. The reduced mobility of palatine pillars and palatine velum during the “A” vowel suggests a more compromised anatomy^45^, which should contribute to oropharyngeal lateral wall obstruction. However, our data has only offered a tendency for such hypothesis. On the other hand, the palatine pillars’ good mobility during the “A” vowel has presented a tendency close to significance to be associated with the tongue obstruction during DISE^46^. A more retrusive profile with a smaller mandible should contribute to a smaller airway leading to tongue obstruction^47^. In our study, this facial feature has had a tendency not only with the tongue base obstruction but also with the obstruction at the oropharyngeal lateral wall, a piece of data another study had already found^48^.

In a study using a CT scan^49^ to measure the soft palate length (from the posterior nasal spine to the uvula tip) length was correlated with the velopharyngeal collapse^49^. Nonetheless, assessing the soft palate length visually and rating subjectively showed no correlation in this study. Nevertheless, a systematic review analyzing several morphological measures with OSA has only demonstrated a correlation with the positioning of the hyoid bone, the pharynx’s length, and facial height^50^. Another study using digital morphometry revealed the tongue size as an efficient and economical method to predict OSA^51^, although, in this study, tongue size (volume, width, and height) in patients with normal BMI did not correlate with the upper airway obstruction detected at any site.

Moreover, our data suggest that it may be difficult to determine one specific myofunctional characteristic to predict the site of upper airway obstruction during DISE, and many different anatomical variations detected highlight the multifactorial physiopathology of OSA. To further clarify the possible correlations between a myofunctional assessment and the obstructive site detected during DISE, OSA subjects should be compared with a control group without OSA - but it is difficult to ethically justify an investigation using a test that puts patients without any disease at risk. Considering that this preliminary study has limitations due to the number of subjects, which jeopardizes more consistent statistical analysis, more studies with similar assessment instruments must be performed to confirm our findings.

This is the first study associating such data, introducing a new comprehensive method of awake upper airway evaluation, and it certainly is worth trying this type of assessment to predict the upper airway obstructive site in OSA. The myofunctional assessment shares the same base as the orofacial myofunctional SLP approach, considering the structures/musculature that can be organized with isotonic and isometric exercises and functions. The detailed assessment helps the SLP fulfills the work with no need for complementary assessments when referring to other professionals. If proven to be reliable, the description of anatomic characteristics that can be associated with the upper airway collapse site may help guide future exercise protocols and other treatment decision options.

## CONCLUSION

This is a preliminary study using an SLP evaluation for sleep-disordered breathing. The instrument with questionnaires and myofunctional orofacial and oropharyngeal assessment to OSA patients was presented and correlated to the upper airway obstructive site detected during DISE.

Higher snoring frequency and intensity, measured with the Berlin questionnaire, was observed in patients with obstruction at the velopharynx and oropharyngeal lateral wall. Subjects with obstruction at the velopharynx had poor tongue sucking ability and sustain it within the hard palate. Obstruction at the epiglottis was the variable that presented many structural and functional associations - including the structures evaluated of the oropharyngeal lateral wall, affected by the palatine tonsils size, depth and width of the hard palate, tongue position, and flaccidity of the floor of mouth.

Although the association between the awake evaluation and DISE has not been conclusive, further research based on this study may lead to interesting new discoveries.
